# Sex differences in audience effects on anogenital scent marking in the red-fronted lemur

**DOI:** 10.1038/s41598-022-08861-2

**Published:** 2022-03-28

**Authors:** Louise R. Peckre, Alexandra Michiels, Lluís Socias-Martínez, Peter M. Kappeler, Claudia Fichtel

**Affiliations:** 1 Behavioral Ecology & Sociobiology Unit, German Primate Center GmbH–Leibniz Institute for Primate Research, Göttingen, Germany; 2 Cognitive Ethology Lab, German Primate Center GmbH–Leibniz Institute for Primate Research, Göttingen, Germany; 3 Institute of Forest Growth and Forest Computer Sciences, Technical University of Dresden, Dresden, Germany; 4 Department Sociobiology/Anthropology, Johann-Friedrich-Blumenbach Institute of Zoology and Anthropology, University of Göttingen, Göttingen, Germany; 5 Leibniz Science Campus Primate Cognition, Göttingen, Germany

**Keywords:** Evolution, Zoology

## Abstract

How the presence of conspecifics affects scent mark deposition remains an understudied aspect of olfactory communication, even though scent marking occurs in different social contexts. Sex differences in scent-marking behaviour are common, and sex-specific effects of the audience could therefore be expected. We investigated sex differences in intra-group audience effects on anogenital scent marking in four groups of wild red-fronted lemurs (*Eulemur rufifrons*) by performing focal scent-marking observations. We observed a total of 327 events divided into 223 anogenital scent-marking events and 104 pass-by events (i.e. passage without scent marking). Using a combination of generalised linear mixed models and exponential random graph models, we found that scent marking in red-fronted lemurs is associated with some behavioural flexibility linked to the composition of the audience at the time of scent deposition. In particular, our study revealed sex differences in the audience effects, with males being overall more sensitive to their audience than females. Moreover, we show that these audience effects were dependent on the relative degree of social integration of the focal individual compared to that of individuals in the audience (difference in Composite Sociality Index) as well as the strength of the dyadic affiliative relationship (rank of Dyadic Composite Sociality Index within the group). The audience effects also varied as a function of the audience radius considered. Hence, we showed that scent marking in red-fronted lemurs is associated with some behavioural flexibility linked to the composition of the audience, ascribing red-fronted lemurs’ social competence in this context.

## Introduction

The traditional approach of considering communication as information transfer between a sender-receiver dyad connected by a transmission channel^1^ has been extended by the concept of communication networks. Indeed, in many social groups, individuals are closely spaced, and signals reach multiple individuals, including both intended and unintended receivers^2–4^. Unintended receivers, i.e. eavesdroppers, can exploit information to their benefit, sometimes at a cost to the sender^3,5^. Accordingly, senders may be sensitive to the presence and characteristics of receivers and may, thus, exhibit behavioural flexibility by initiating, inhibiting, or varying the rate or nature of signal deposition^6^. Such effects are defined as ‘audience effects’^6,7^.

Although olfactory signals represent a main modality of communication in most mammals^5,8^, audience effects have mainly been studied for vocal and visual signals^7^. This inbalance can be explained by different reasons. First, olfactory signals are long-lasting, remaining in the environment long after the sender left the location. Hence, these signals may be perceived even in the absence of an audience at the time of their deposition. Second, historically, research on olfactory communication focused mainly on solitary species, where audience effects on the deposition of olfactory signals appeared to be less relevant^7^. However, scent signals have now been shown to be deposited in many different social contexts and are recognised as important in social species both for within- and between-group communication^9–14^. Moreover, recent frameworks highlighted the importance of selective pressures arising from the social domain on the evolution of communicative systems across all modalities^15,16^.

Interestingly, scent-marking behaviours, defined as motor patterns used to deposit chemical secretions or excretions (e.g., urine, saliva, anogenital secretions) on objects or conspecifics^17–19^, often take the form of conspicuous ephemeral visual displays^20^. These visual components might immediately attract the attention of individuals present in the vicinity and guide them to the signal’s long-lasting olfactory component. Hence, this multimodal nature may confer scent-marking behaviour the capacity to be addressed both to the audience present during deposition and to unknown future receivers^5,21,22^. The idea of a chemical component deposited using a conspicuous visual display that would attract the individuals present in the audience was formalised under the ‘demonstrative marking hypothesis’. This hypothesis was postulated for territorial male Thomson’s gazelles (*Eudorcas thomsonii*), which combine urine-faeces deposition with an extreme body posture display^20,23,24^. Palagi and Norscia^25^ also described such a ‘composite effect’ in ring-tailed lemurs (*Lemur catta*), which either urinate with the tail only slightly raised or combine urine-marking with a conspicuous visual signal, the up-right erection of the tail. The erection of their tail attracted the attention of receivers to the location of the urine deposition, and resulted in more group members inspecting the urine-mark compared to urine-marks deposited without this tail display^25^. However, to date, how the composition of the audience may affect scent deposition remains an understudied aspect of olfactory communication.

Scent marks can carry reliable information about the sender’s age, sex, health, reproductive and social status^26–32^. Scent-marking behaviour has been associated with various functions, both across^33–36^ and within species^21,37–39^. They can be classified into three broad functional categories: sexual attraction, competition, and parental care^22,33–36^. Functional differences in scent-marking behaviour between the sexes have been described in numerous species (e.g. mandrills *Mandrillus sphinx*^31^, moustached tamarins *Saguinus mystax*^40^, cheetahs *Acinonyx jubatus*^39^, giant pandas *Ailuropoda melanoleuca*^41^, honey badgers *Mellivora capensis*^42^). These functional sex differences are commonly associated with morphological, physiological and behavioural differences^14,21,28,36,39,43–50^. Considering these functional differences between the sexes, sex-specific effects of the audience when depositing scent marks can be expected.

Strepsirrhines primates, like most other mammals, have a functional vomeronasal organ^51^. They rely heavily on olfactory communication and produce a wide variety of scent signals expressed by glands located in various body areas (i.e. head, neck, chest, forelimb and anogenital area)^14,22,52,53^. Among strepsirrhines, true lemurs (i.e. genus *Eulemur*, Lemuridae) include nine species with comparable glands in their genital and perianal regions. In these species, anogenital scent marking is relatively frequent and occurs across different contexts^16^. Anogenital scent marks in true lemurs have been shown to carry information on species identity^14,54^, phylogeny^14,16^, social system^14^, sex^16,55^, odorant source^14,16^, individuality^56^ and reproductive state^16^. Morphological and physiological sex differences associated with anogenital scent marking also exist in true lemurs. First, females have more elaborated anogenital glands than males^16^. Second, the chemical richness of genital secretions differs between the sexes as a function of a species’ social structure. In female-dominant species, chemical richness is higher in females, while in species without overt dominance relationships between sexes, chemical richness is higher in males^16^. Moreover, while previous studies reported no sex difference in the average frequency of anogenital scent marking^57,58^, studies addressing this question are scarce and all carried out in captivity leaving the question open.

We investigated audience effects on anogenital scent-marking behaviour in wild red-fronted lemurs (*Eulemur rufifrons*). Red-fronted lemurs live in cohesive small multi-female–multi-male groups of 5–12 individuals with an even or male-biased adult sex ratio^59–63^. They are promiscuous, with all females mating with almost all males within their group^64^ and do not exhibit strong male–female bonds^65^. They lack clear intersexual dominance relationships, with neither sex being consistently dominant over the other^65,66^ and aggression rates are low within both sexes^67^. However, one male (referred to as central male) in each group seems to be involved in more social interactions with all females than all other males^65^. Central males sire around 60–70% of all infants^61,68^ and scent-mark more than other males in the group^65^. Among females competition can be intense, with females evicting even related females from the group when they reach a critical group size^69^.

In this study, we examined whether males and females differed in their sensitivity to an audience when anogenital scent marking. In principle, it is challenging to define the potential audience in animals because the attention to signals may depend on the distance between the sender and potential receivers in the audience. Moreover, if senders differentiate between the composition of the audience in proximity and overall presence of individuals in the broader audience, different audience effects can be observed depending of the audience radius considered. In an earlier study on red-fronted lemurs, Sperber and colleagues^63^ have shown that collective-decision making during group departure depends on the inter-individual distance between initiators and followers, with individuals being closer to the initiator following them more readily. We, therefore, chose the same distances (3, 5, and 10 m) to define different categories of audience. Red-fronted live in a forest environment where visibility rapidly decreases with distance. However, as we were able ourselves to assess the audience composition until ten meters reliably, decreased visibility is unlikely to impact the animals’ perception of the audience composition in any of the chosen ranges.

We predict (1) the presence of audience effects on anogenital scent marking in red-fronted lemurs. These audience effects are expected to (2) vary between the sexes and to (3) be dependent on the relative degree of social integration of the focal individual compared to that of individuals in the audience (difference in Composite Sociality Index^70^, hereafter difference in CSI) as well as the strength of the dyadic affiliative relationship (rank of Dyadic Composite Sociality Index^71^ within the group, hereafter DSI rank). The audience effects are also predicted to (4) vary depending on the audience radius considered.

## Results

We identified 177 scent-marking spots, defined as a place where we observed at least one individual scent marking anogenitally. At these scent-marking spots, we observed a total of 327 events consisting of 223 anogenital scent-marking events (105 in males and 118 in females) and 104 pass-by events (i.e. passage without scent marking; 60 in males and 44 in females).

**Figure 1:**
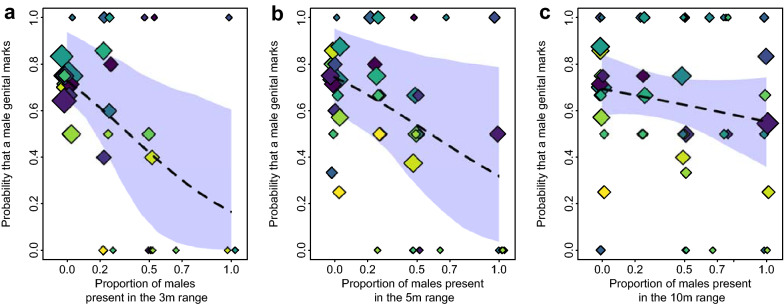
Probability that a male deposited a scent mark depending on the proportion of males present (**a**) in a 3 m radius, (**b**) in a 5 m radius, (**c**) in a 10 m radius. Colours correspond to the different individuals (n = 14), and the size of the circle corresponds to the number of observations (in total, n = 165). This figure was visualized and edited using R (https://www.r-project.org/).

In males, we found a significant audience effect within the 3 m radius (full-null model comparison: χ^2^ = 6.48, df = 2, p = 0.039; R^2^_m_ = 0.10, R^2^_c_ = 0.23, n_mark_ = 105 and n_pass_ = 60). Notably, males anogenital-marked less often when a higher proportion of males were present (χ^2^ = 6.23, df = 1, p = 0.013, p_adjusted_ = 0.039, Table 1, Fig. 1a). When removing the cases in which no males were present in the audience, this relationship persisted (χ^2^ = 4.91, df = 1, p = 0.027, n_mark_ = 29 and n_pass_ = 25). However, this audience effect was detected only by trend in the 5 m radius and not in the 10 m radius (full-null model comparisons: for 5 m, χ^2^ = 4.77, df = 2, p = 0.092, Fig. 1b; for 10 m, χ^2^ = 1.47, df = 2, p = 0.481, Table 1, Fig. 1c). In addition, for all three distance radii, neither the proportion of females, age of the focal male (adult or subadult), context (group activity defined as resting, feeding, travelling or disturbance), nor season significantly affected the probability of anogenital marking in males (Table 1).

**Table 1:** Results of the models of the effects of audience composition within 3, 5 and 10 m radius, age, context and season on the probability that a male scent-mark when passing a marking spot.

	Audience radius	Estimate	Standard errors	Lower CI	Upper CI	Chi-squared	df	p-value	Minimum	Maximum
Intercept	3 m	0.26	0.51	− 0.94	1.64	–	–	–	− 0.13	0.56
	5 m	0.73	0.55	− 0.58	2.72				0.36	1.09
	10 m	0.80	0.63	− 0.27	1.94				0.34	1.34
Proportion of males in the audience	3 m	− 2.74	1.02	− 7.65	− 0.83	6.23	1	**0.013**	− 3.64	− 2.19
	5 m	− 1.83	0.84	− 5.11	− 0.21	4.55	1	**0.033**	− 2.37	− 0.93
	10 m	− 0.83	0.64	− 1.88	0.65	1.18	1	0.277	− 1.34	− 0.37
Proportion of females in the audience	3 m	1.77	1.06	− 0.02	5.71	2.81	1	0.094	1.13	2.82
	5 m	0.40	0.78	− 1.60	2.73	0.20	1	0.658	− 0.06	1.06
	10 m	0.06	0.77	− 1.32	1.35	0.00	1	0.995	− 0.50	0.41
Age—sub-adult	3 m	− 0.21	0.53	− 0.64	1.25	0.10	1	0.341	− 0.55	0.21
	5 m	0.21	0.66	− 1.18	1.92	0.04	1	0.848	− 0.38	0.69
	10 m	− 0.15	0.52	− 1.25	0.69	0.42	1	0.515	− 0.48	1.13
Context—disturbance	3 m	0.94	0.79	− 0.45	4.65	1.53	3	0.795	0.50	1.48
	5 m	0.62	0.59	− 0.81	3.65	1.60	3	0.659	0.37	1.16
	10 m	0.77	0.61	− 0.14	2.42	2.60	3	0.457	0.41	1.18
Context—resting	3 m	0.27	0.88	− 2.43	4.85	1.53	3	0.795	− 0.25	1.37
	5 m	0.13	1.72	− 6.95	11.01	1.60	3	0.659	− 0.48	5.54
	10 m	0.10	1.14	− 1.28	1.95	2.60	3	0.457	− 0.52	5.96
Context—traveling	3 m	0.33	0.57	− 0.98	1.97	1.53	3	0.795	0.00	0.81
	5 m	− 0.09	0.57	− 1.86	1.52	1.60	3	0.659	− 0.34	0.37
	10 m	− 0.08	0.56	− 0.88	1.12	2.60	3	0.457	− 0.45	0.26
Season—mating	3 m	0.29	0.67	1.49	2.96	0.12	1	0.511	0.02	0.81
	5 m	0.38	0.78	1.68	4.43	0.13	1	0.714	0.02	0.70
	10 m	0.04	0.70	1.42	1.26	0.01	1	0.914	0.47	0.51

Significant p-values are in bold.

In females, we found no audience effect associated with the proportion of individuals present (full-null model comparison: for 3 m, χ^2^ = 4.84, df = 2, p = 0.089; for 5 m, χ^2^ = 6.85, df = 2, p = 0.032; for 10 m χ^2^ = 3.54, df = 2, p = 0.171; Table 2). Neither the proportion of males, the proportion of females, age, context, nor season predicted the probability of anogenital scent marking (Table 2).

**Table 2:** Results of the models of the effects of audience composition within 3. 5 and 10 m radius, age, context and season on the probability that a female scent− marked when passing a marking spot.

	Audience radius	Estimate	Standard errors	Lower CI	Upper CI	Chi− squared	df	p− value	Minimum	Maximum
Intercept	3 m	1.41	0.39	0.71	2.44	–	–	–	− 0.17	0.48
	5 m	1.61	0.45	0.89	2.99				1.46	2.00
	10 m	1.95	0.68	0.99	5.00				1.68	2.40
Proportion of males in the audience	3 m	− 0.67	0.84	− 2.62	1.34	0.64	1	0.425	− 3.67	− 2.12
	5 m	− 0.50	0.85	− 2.49	1.35	0.34	1	0.558	− 1.05	− 0.07
	10 m	− 1.75	1.00	− 5.45	− 0.06	3.01	1	0.083	− 2.67	− 1.34
Proportion of females in the audience	3 m	− 0.71	0.71	− 2.50	0.78	1.00	1	0.319	0.95	2.79
	5 m	− 0.95	0.66	− 2.60	0.48	2.13	1	0.145	− 1.29	− 0.55
	10 m	0.23	0.73	− 1.48	2.45	0.11	1	0.745	0.03	0.96
Context—disturbance	3 m	− 0.10	0.53	− 1.20	1.23	1.01	3	0.799	0.11	0.76
	5 m	− 0.09	0.53	− 1.24	1.27	1.30	3	0.730	− 0.44	0.17
	10 m	− 0.07	0.56	− 1.73	2.05	1.71	3	0.634	− 0.35	0.30
Context—resting	3 m	0.34	0.64	− 0.92	2.75	1.01	3	0.799	0.35	1.36
	5 m	0.18	0.65	− 1.30	2.53	1.30	3	0.730	− 0.14	0.92
	10 m	0.55	0.79	− 1.11	6.57	1.71	3	0.634	0.21	1.44
Context—traveling	3 m	− 0.30	0.49	− 1.44	0.80	1.01	3	0.799	− 0.37	1.45
	5 m	− 0.47	0.51	− 1.79	0.64	1.30	3	0.730	− 0.81	− 0.26
	10 m	− 0.55	0.61	− 2.65	0.91	1.71	3	0.634	− 0.92	− 0.29
Season—mating	3 m	− 0.13	0.48	− 1.18	1.29	0.07	1	0.790	− 0.03	0.73
	5 m	− 0.02	0.49	− 1.08	1.35	0.00	1	0.962	− 0.29	0.22
	10 m	− 0.14	0.59	− 1.69	2.21	0.06	1	0.813	− 0.38	0.16

When considering the anogenital-marking network (exponential random graph model), overall, sex and/or sociality (i.e. DSI rank of the dyad focal-audience, difference in the CSI values of the individuals within a given dyad, CSI of the individual in the audience) had an effect on the probability of an individual to scent mark in front of another individual (full-null model comparison: 3 m chi^2^ = − 697.5, df = 580, p < 0.001, p_adjusted_ < 0.001; 5 m chi^2^ = − 1263.1, df = 580, p < 0.001, p_adjusted_ < 0.001; 10 m chi^2^ = − 1982.8, df = 580, p < 0.001, p_adjusted_ < 0.001).

**Figure 2:**
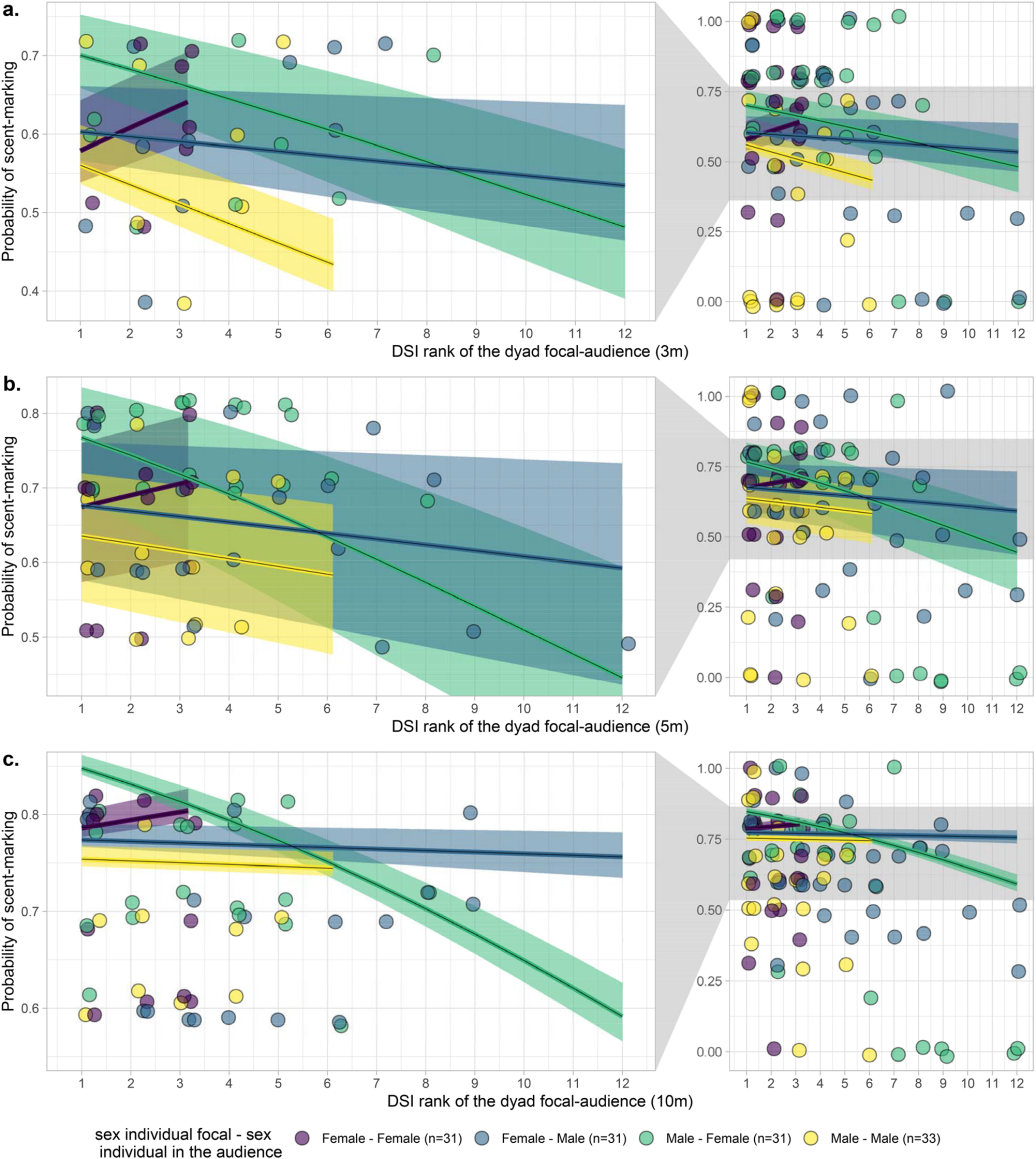
Probability to scent-mark as a function of the DSI rank of the dyad for an audience (**a**) in a 3 m radius,(**b**) in a 5 m radius, and (**c**) in a 10 m radius. The shaded areas show 95% confidence intervals of the model (conditional on the number of observations being at its average and on a group effect weighted by the number of individuals in each group). The first column of plots presents the zoomed-in section of the full range, indicated by grey polygons, to better depict the corresponding regression lines. This figure was visualized and edited using R (https://www.r-project.org/).

In particular, there was a significant effect of the interaction between the combination of sexes and the DSI rank of the respective dyad on the probability of scent marking within the 5 m and 10 m radius (full-reduced model comparison: 5 m chi^2^ = − 1412.5, df = 568, p-value = 0.003, p_adjusted_ = 0.010; 10 m chi^2^ = − 2213.5, df = 568, p-value < 0.001, p_adjusted_ < 0.001; Table 3) but not within the 3 m radius (chi^2^ = − 749.5, df = 568, p-value = 0.184, p_adjusted_ = 0.550). Males scent marked more often in front of females with whom they had a stronger relationship (smaller DSI rank). In contrast, females scent-marked more often in front of females with whom they had a weaker relationship (higher DSI rank) (Fig. 2).

**Table 3:** Results of the exponential graph model of the effects of audience composition within 3, 5 and 10 m radius on the probability that an individual scent-mark when passing a marking spot.

	Audience radius	Estimate	Standard errors	z-value	Chi-squared	df	p-value
Sum	3 m	1.017	0.327	3.112			**0.002**
	5 m	1.678	0.269	6.228			** < 0.001**
	10 m	2.171	0.227	9.544			** < 0.001**
Nonzero	3 m	− 1.542	0.423	− 3.648			** < 0.001**
	5 m	− 1.713	0.546	− 3.134			**0.002**
	10 m	− 1.398	0.664	− 2.106			**0.035**
Mutual	3 m	0.357	0.161	2.212			**0.027**
	5 m	0.261	0.137	1.908			0.056
	10 m	0.168	0.115	1.456			0.145
Cyclical weights	3 m	0.146	0.072	2.028			**0.043**
	5 m	0.150	0.079	1.897			0.058
	10 m	− 0.005	0.064	− 0.074			0.941
Female adult^b^	3 m	0.017	0.344	0.048			^a^
	5 m	− 0.220	0.288	− 0.765			
	10 m	0.130	0.227	0.574			
Male adult^b^	3 m	− 0.623	0.287	− 2.166			^a^
	5 m	− 0.520	0.221	− 2.352			
	10 m	− 0.077	0.186	− 0.411			
Male when female in the audience^c^	3 m	− 0.068	0.294	− 0.230			^a^
	5 m	0.227	0.261	0.867			
	10 m	0.186	0.197	0.943			
Female when male in the audience^c^	3 m	0.141	0.284	0.494			^a^
	5 m	0.332	0.212	1.564			
	10 m	0.205	0.164	1.250			
Female when female in the audience^c^	3 m	− 0.615	0.318	− 1.931			^a^
	5 m	− 0.295	0.268	− 1.100			
	10 m	− 0.165	0.204	− 0.809			
CSI rank of the individual in the audience	3 m	0.084	0.092	0.916			^a^
	5 m	0.021	0.073	0.289			
	10 m	− 0.027	0.054	− 0.501			
CSI rank of female in the audience^d^	3 m	0.181	0.150	1.207	− 785.8^f^	566^f^	**0.003**^**f**^
	5 m	0.287	0.124	2.317	− 1399.6^f^	566^f^	0.125^f^
	10 m	0.213	0.098	2.163	− 2120.3^f^	566^f^	** < 0.001**^**f**^
Difference in the CSI	3 m	0.081	0.108	0.746			
	5 m	0.167	0.088	1.896			
	10 m	0.003	0.068	0.045			
Difference in the CSI (female focal–female in audience)^c^	3 m	− 0.130	0.181	− 0.719	− 768.7^f^	568^f^	**0.044**^**f**^
	5 m	− 0.120	0.151	− 0.794	− 1312.5^f^	568^f^	** < 0.001**^**f**^
	10 m	− 0.072	0.124	− 0.584	− 2208.8^f^	568^f^	** < 0.001**^**f**^
Difference in the CSI (male focal–female in audience)^c^	3 m	− 0.146	0.256	− 0.570	− 768.7^f^	568^f^	**0.044**^**f**^
	5 m	− 0.174	0.204	− 0.856	− 1312.5^f^	568^f^	** < 0.001**^**f**^
	10 m	− 0.007	0.161	− 0.045	− 2208.8^f^	568^f^	** < 0.001**^**f**^
Difference in the CSI rank (female focal–male in audience)^c^	3 m	0.033	0.192	0.174	− 768.7^f^	568^f^	**0.044**^**f**^
	5 m	− 0.151	0.163	− 0.931	− 1312.5^f^	568^f^	** < 0.001**^**f**^
	10 m	− 0.094	0.128	− 0.734	− 2208.8^f^	568^f^	** < 0.001**^**f**^
DSI rank	3 m	− 0.190	0.158	− 1.200			^a^
	5 m	− 0.082	0.126	− 0.652			
	10 m	− 0.018	0.100	− 0.182			
DSI rank (female focal–female in audience)^c^	3 m	0.419	0.313	1.340	− 737.3^f^	568^f^	** < 0.001**^**f**^
	5 m	0.221	0.266	0.831	− 1393.5^f^	568^f^	**0.037**^**f**^
	10 m	0.115	0.206	0.558	− 2206.2^f^	568^f^	** < 0.001**^**f**^
DSI rank (male focal–female in audience)^c^	3 m	0.030	0.189	0.158	− 737.3^f^	568^f^	** < 0.001**^**f**^
	5 m	− 0.162	0.152	− 1.068	− 1393.5^f^	568^f^	**0.037**^**f**^
	10 m	− 0.215	0.123	− 1.739	− 2206.2^f^	568^f^	** < 0.001**^**f**^
DSI rank (female focal–male in audience)^c^	3 m	0.141	0.178	0.792	− 737.3^f^	568^f^	** < 0.001**^**f**^
	5 m	0.019	0.142	0.136	− 1393.5^f^	568^f^	**0.037**^**f**^
	10 m	0.001	0.112	0.012	− 2206.2^f^	568^f^	** < 0.001**^**f**^
Group B^e^	3 m	0.038	0.079	0.487	− 703.6^f^	568^f^	** < 0.001**^**f**^
	5 m	− 0.074	0.065	− 1.141	− 1257.4^f^	568^f^	** < 0.001**^**f**^
	10 m	− 0.184	0.056	− 3.303	− 2103.1^f^	568^f^	** < 0.001**^**f**^
Group F^e^	3 m	0.210	0.087	2.421	− 703.6^f^	568^f^	** < 0.001**^**f**^
	5 m	0.198	0.067	2.956	− 1257.4^f^	568^f^	** < 0.001**^**f**^
	10 m	0.061	0.056	1.087	− 2103.1^f^	568^f^	** < 0.001**^**f**^
Group J^e^	3 m	− 0.262	0.088	− 2.994	− 703.6^f^	568^f^	** < 0.001**^**f**^
	5 m	− 0.359	0.072	− 5.012	− 1257.4^f^	568^f^	** < 0.001**^**f**^
	10 m	− 0.420	0.059	− 7.101	− 2103.1^f^	568^f^	** < 0.001**^**f**^
Number of times the individual was observed when a given individual was in the audience	3 m	0.783	0.089	8.845			** < 0.001**
	5 m	0.645	0.070	9.165			** < 0.001**
	10 m	0.494	0.055	9.039			** < 0.001**

Significant p-values are in bold.

^a^Not shown because of having a very limited interpretation.

^b^Comparisons with the reference level (male sub-adult).

^c^Comparisons with the reference level (male focal–male in audience).

^d^Comparisons with the reference level (male in the audience).

^e^Comparisons with the reference level (Group A).

^f^Value corresponding to the full-reduced model comparison.

**Figure 3:**
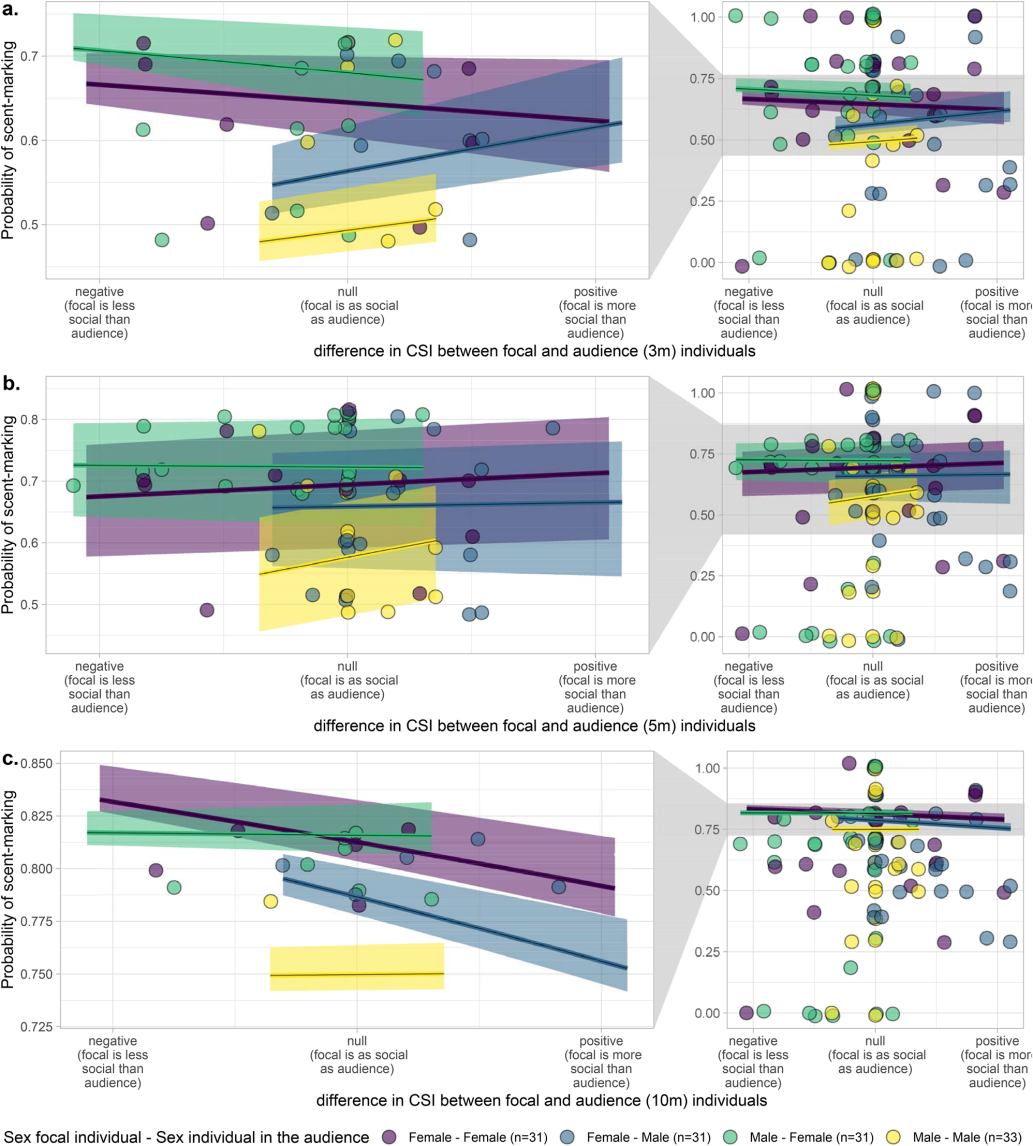
Probability to scent-mark as a function of the difference in CSI of the dyad for an audience (**a**) in a 3 m radius, (**b**) in a 5 m radius, and (**c**) in a 10 m radius. The shaded areas show 95% confidence intervals of the model (conditional on the number of observations being at its average and on a group effect weighted by the number of individuals in each group). The first column of plots presents the zoomed-in section of the full range, indicated by grey polygons, to better depict the corresponding regression lines. This figure was visualized and edited using R (https://www.r-project.org/).

There was a significant interaction effect between the sexes and the CSI difference between individuals of the respective dyad within the 3 m and 10 m radius (full-reduced model comparison: 3 m chi^2^ = − 740.8, df = 568, p-value = 0.004, p_adjusted_ = 0.011; 10 m chi^2^ = − 2225.5, df = 568, p-value < 0.001, p_adjusted_ < 0.001; Table 3) but not within the 5 m radius (chi^2^ = − 1393.5, df = 568, p-value = 0.152, p_adjusted_ = 0.455). Females scent marked more often in front of females that were more social than themselves (i.e. when the difference in CSI was negative; Fig. 3). Females scent-marked more often when males that were as social as themselves (i.e. when the difference in CSI is small) were present in the 10 m range, but their probability to scent-mark also increased when in close proximity (< 3 m) with males that were less social than themselves (i.e. when the difference in CSI is positive; Fig. 3).

**Figure 4:**
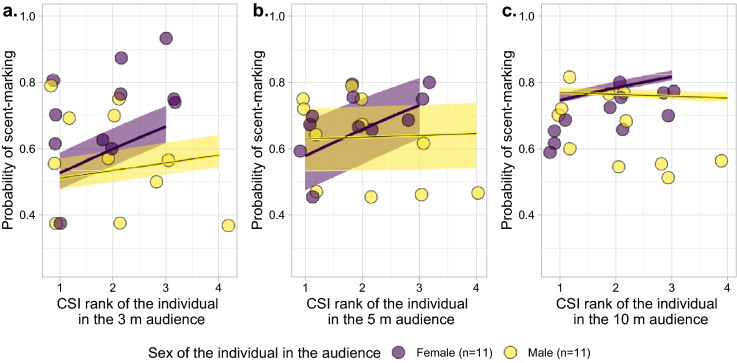
Probability to scent-mark as a function of the CSI rank of the individual in the audience when (**a**) in a 3 m radius, (**b**) in a 5 m radius, and (**c**) in a 10 m radius. The shaded areas show 95% confidence intervals of the model (conditional on the number of observations being at its average and on a group effect weighted by the number of individuals in each group). The first column of plots presents the zoomed-in section of the full range, indicated by grey polygons, to better depict the corresponding regression lines. This figure was visualized and edited using R (https://www.r-project.org/).

There was also a significant interaction effect between sex and CSI rank of the individual in the audience across all distances (full-reduced model comparison: 3 m chi^2^ = − 776.0, df = 566, p-value < 0.001, p_adjusted_ < 0.001; 5 m chi^2^ = − 1351.9, df = 566, p-value < 0.001, p_adjusted_ < 0.001; 10 m chi^2^ = − 2237.0, df = 566, p < 0.001, p_adjusted_ < 0.001; Table 3). More specifically, individuals scent marked more often in front of the less social females (the ones exhibiting a greater CSI rank; Fig. 4).

The probability for an individual to be observed marking in front of a given conspecific increased as a function of the number of times the individual was observed in the presence of this individual (3 m: b = 0.783, p < 0.001, p_adjusted_ < 0.001; 5 m: b = 0.645, p < 0.001, p_adjusted_ < 0.001; 10 m: b = 0.494, p < 0.001, p_adjusted_ < 0.001; Table 3). Hence, individuals were more likely to scent mark in front of individuals that were more often in proximity to them. Moreover, there was a significant effect of the group on the probability to observe scent marking (full-null model comparison: 3 m: chi^2^ = − 729.6, df = 568, p < 0.001, p_adjusted_ < 0.001; 5 m: chi^2^ = − 1222.2, df = 568, p < 0.001, p_adjusted_ < 0.001; 10 m: chi^2^ = − 2122.1, df = 568, p < 0.001, p_adjusted_ < 0.001; Table 2). There was no significant effect of the mutual term after correcting for multiple testing (3 m: b = 0.357, p = 0.027, p_adjusted_ = 0.081; 5 m: b = 0.261, p = 0.056, p_adjusted_ = 0.168; 10 m: b = 0.168, p = 0.145, p_adjusted_ = 0.435; Table 3). There was also no significant effect of the cyclical term after correcting for multiple testing (3 m: b = 0.146, p = 0.043, p_adjusted_ = 0.129; 5 m: b = 0.150, p = 0.058, p_adjusted_ = 0.174; 10 m: b = − 0.005, p = 0.941; p_adjusted_ = 1; Table 3).

## Discussion

In this study, we investigated intra-group audience effects on anogenital scent marking in wild red-fronted lemurs. Our results indicated that scent marking in red-fronted lemurs is associated with some behavioural flexibility linked to the composition of the audience at the time of scent deposition. Moreover, our findings also showed that the nature of the audience effects differed between males and females, with males being more sensitive to their audience than females.

On the intrasexual level, males were observed to scent mark significantly less often when a greater proportion of males of their group were within the 3 m radius. This observation is reinforced by the lowest anogenital scent-marking probabilities associated with the male-male category in the outputs of the exponential random graph analyses (Figs. 2 and 3). However, the effect of the proportion of males present in the audience on the probability of a male to anogenital scent-mark was detected only by trend in the 5 m radius and absent in the 10 m radius). In principle, it is possible that the individuals present in the 3 to 10 m range of the scent-marking spot were too far away to be attentive to scent mark deposition of other individuals. However, scent-marking rates were predicted by the strength of the social relationship with the individuals in these larger distance categories, suggesting that individuals even when they were farther away might still be attentive to scent-mark depositions. Indeed, the probability that a male would scent mark in front of another male decreased when these two males had a weaker social relationship (greater DSI rank). Males also tended to scent mark less often in front of males that were more social than themselves (negative values of the difference in CSI). Hence, males seem to avoid scent marking in close proximity with an increased number of males, especially if the latter are more social than themselves, and in the presence of males with whom they have weak affiliation.

Therefore, it is possible that even if there is no linear hierarchy and low aggression levels in male red-fronted lemurs^65^, the risk of physical aggression might be elevated when scent marking in close proximity. It might also be that males inhibit their scent-marking behaviour to avoid their scent mark being quickly overmarked by other males^11^. In addition, the probability of the sender receiving an aggression and/or overmarking might be higher when the male in the audience is not a close affiliate and is more central than the scent marker. Investigations on the probability of exhibiting aggression and overmarking at different distances and depending on the social value of the relationship between two individuals might help to test this prediction. Alternatively, males may give priority to other males to scent mark the spot when they are in proximity and prefer to pass by the scent-marking spot without depositing a scent mark. Hence, competition among males may result in having priority of access to these specific scent-marking spots. If the male to whom the priority would be given is at a distance of 5 or 10 m, the focal male might still have time to scent mark before its arrival. Our results indicate that priority of scent marking seems to be given to the most social males, which may also contribute towards explaining why the central males have been observed to be the ones scent marking more frequently^65^. Hence, in red-fronted lemurs, as suggested in an earlier study^65^, males might use anogenital scent marking as a way to advertise their social status to other males and as an indirect form of competition. This function of scent marking has also been suggested for several other lemur species (e.g. ring-tailed lemurs^8,72^, Verreaux's sifaka^38,73–75^; red lemurs *Eulemur rufus*^46^; Milne-Edward's sifakas *Propithecus edwardsi*^76,77^*;* silky sifakas *P. candidus*^76^; grey mouse lemurs *Microcebus murinus*^78^) and other mammalian species^45^ (e.g. brown bears, *Ursus arctos*^79^, house mice *Mus musculus*^80^).

Females were observed to scent-mark more often in the presence of females that were more social than themselves (negative values of the difference in CSI) in the 10 m radius. This effect was not significant when looking at smaller radii, suggesting that females give more importance to the overall audience than to proximity in this context. Females were also observed to scent-mark more often in the presence, at any distance range, of females with whom they had weaker social relationships (greater DSI rank). Hence, despite the absence of hierarchical dominance instigated through overtly aggressive behaviour, females may signal their social status via scent marks. However, the highest scent-marking probabilities are associated with the female-female category in the outputs of the exponential random graph analyses (Figs. 2, 3), suggesting that overall, females seem to be less sensitive to the presence of females in their audience than to the presence of males.

On the intersexual level, we found no effect of the proportion of individuals of one sex present in the audience on the probability of an individual of the opposite sex to anogenital scent mark. However, females were observed to increase their scent-marking probability when in close proximity (< 3 m) with males that were less social than themselves (positive values of the difference in CSI). When considering a 10 m radius females were observed to scent mark more often when males that were as social as themselves (i.e. when the difference in CSI is small) than when males that were less social than themselves were present, suggesting that the proximity of the males has an effect on a female’s decision to scent-mark. Females may generally prefer to scent mark in front of the most socially integrated males of the group but may also address their scent mark to the less integrated males when in close proximity with them (personal observations).

Males were observed to scent mark more in the presence of a female when they had a stronger relationship with that female (lower DSI rank). This effect was highly significant at 5 and 10 m, showing that the presence of such females was more important than her proximity to the focal male. Hence, males may particularly address scent-mark signals to females with whom they maintain a close relationship. This outcome is in line with earlier research suggesting that males that are involved in more social interactions with females than all other males (i.e. central males) are the ones scent marking the most^65^.

At the intersexual level, scent-marking behaviours may serve to maintain the pair-bonding, as shown in both pair-living (red-bellied lemurs *Eulemur rubriventer*^81^) and group-living species (Coquerel’s sifakas *Propithecus coquereli*^82^). Scent-marking signals have also been suggested to be directed towards the opposite sex as a form of mate attraction (ring-tailed lemurs^83^ and grey mouse lemurs^78^). Both functions are not contradicted by our results but further research on the function of scent-marks is required.

Overall, our results indicate that males seem to be more sensitive than females to their audience when scent marking. Whereas both males and females seem to be sensitive to the audience, social facilitation of scent marking may occur in females, whereas in males social inhibition of scent marking may occur. Social facilitation and inhibition are defined respectively as an increase or decrease of the initiation, frequency or intensity of a response in the presence of other individuals^84–87^. Hence, males seem to be more constrained in the expression of scent signals and appear to adjust their scent-marking behaviour in a more fine-tuned manner to the composition of the audience than females. Less social males, which scent mark less frequently in the presence of other males, may rely primarily on the long-lasting component of the signal to advertise their social status to a future audience, thereby avoiding potential aggression from other males.

Interestingly, male genital secretions have been shown to be chemically richer than the genital secretions of females in true lemur species without overt dominance relationships^14,16^. Social constraints on signal deposition may be balanced by a more elaborate signal design in these species. Studying the flexibility of multicomponent signal usage across social contexts (audience compositions) contributes to uncovering the social features eliciting or constraining complex signal expression^15,88^. These social characteristics may, in turn, constitute social pressures acting for or against the evolution of complex signalling behaviour^6,15,22,89^. Moreover, in true lemurs, diversification of means of olfactory communication covaried with the diversification of social systems, making them excellent models for comparative studies in this context^16,53^. Hence, further research combining chemical analyses with observations of scent-marking behaviour and audience effects across true lemur species are now indicated to further understand the social function of scent-marking behaviours.

The term social facilitation is used both in the case when the other individuals are engaged in a similar task or behaviour (co-action) or when they are passive observers (restrictive use of the term “audience effect”)^9,84–87^. In the present study, the individual scent marking did neither always observe another individual scent marking nor pass in proximity to a recently deposited scent mark. Indeed, this is the case for the first event of almost all video recordings as we started recording the individuals before observing a scent-marking event. Moreover, the 15 min duration of the focal observations, allowed for individuals or sub-groups isolated from the rest of the group to also perform behaviours without having been part of the audience of an individual recorded earlier. For these reasons, social facilitation via co-action is unlikely in the context of this study.

Social facilitation via co-action historically implies arousal-mediated mechanisms, while the ‘audience effect’ sometimes refers to the specific effect that an individual is being watched or thinks it is being watched^86,90^. Audience effects may indeed reveal a potential intentional communication, primarily when this variation is based on subtle social and behavioural variations, such as the quality of relationships^6,90–93^. Here we show that red-fronted lemurs do not only scent mark flexibly as a function of the proportion of males and females present in the audience but also based on the strength of the social relationship they maintain with specific individuals present in the audience. Such social competence was described as one indicator of potential intentionality in signalling behaviour^90,94^.

Finally, some caveats and limitations of our study need to be mentioned. First, some individuals may also choose not to pass a specific scent-marking spot in the presence of a particular audience. Hence, we cannot exclude and control for a potential audience effect on the probability to pass this spot or not. Second, the effect of who may have marked beforehand on a specific spot might also be relevant in an individual's choice to mark or not when passing a spot. This aspect is difficult to control in the field because we do not have information on the possible passage on this spot before the observations and video recordings started. Further studies on the patterns of scent-marking behaviour succession occurring on a given scent-marking spot may clarify these questions^8^. Moreover, considering the orientation (e.g. facing or facing away the scent-marking spot) of the individuals in the audience may also be an interesting perspective in this regard. While at 3 m, the individuals may be relatively homogeneously attentive to the scent marking of an individual, when further apart they may notice the scent-marking behaviour only when they are facing the scent-marking-spot. As a consequence, individuals approaching the scent-marking spot may indeed be more attentive than individuals that already overpassed this spot. This may also contribute to the lack of some audience effects observed at larger distances.

Besides intra-group functions, scent marking may also be a form of inter-group communication in resource or territorial defence through individual or group odour deposition^49,95–97^. Female red-fronted lemurs are philopatric and remain in the territory of their mother, so they might be motivated to defend their territory and/or its associated resources. As some of the events reported here occurred in the context of post or pre-inter group encounters (with no extra-group individuals in the audience), they could have impacted our results. However, the effect of context did not influence scent mark deposition. Still, exploring in more detail inter-group audience effects may reveal interesting complementary information to understand further how red-fronted lemur flexibly adapt their behaviour to the social context.

In conclusion, we showed that scent marking in red-fronted lemurs is associated with some behavioural flexibility linked to the composition of the audience (i.e. proportion and social value of the individuals present), ascribing red-fronted lemurs social competence in this context. Moreover, our approach broadens our understanding of signal delivery and its associated sex differences in red-fronted lemurs, providing an avenue for future research addressing the question of the effect of social variation on scent-marking behaviour.

## Material and methods

### Study site and subjects

We conducted this study in Kirindy Forest, a dry deciduous forest located ca. 60 km north of Morondava, western Madagascar, managed within a forestry concession operated by the Centre National de Formation, d'Etudes et de Recherche en Environnement et Foresterie (CNFEREF)^98^. Since 1996, all members of a local population of red-fronted lemurs inhabiting a 80-ha study area within the forest have been regularly captured, marked with individual nylon or radio collars, and subjected to regular censuses and behavioural observations as part of a long-term study^98^. The data presented in this study were collected from May to November 2018 on 28 individuals belonging to four groups (11 females and 17 males; Table 4). Among males, 14 were adults and 3 sub-adults (1.5–2 years). Sub-adults were included in the study as they were observed to perform scent-marking behaviour as often as adult individuals. Reproduction of the species is seasonal, with a 4-week mating season in May–June and a birth season in September–October^65,99^. All applicable international, national, and/or institutional guidelines for the care and use of animals were followed. The authors complied with the ARRIVE guidelines^100^. This study adhered to the Guidelines for the Treatment of Animals in Behavioral Research and Teaching^101^ and the legal requirements of the country (Madagascar) in which the work was carried out. The protocol for this research was approved by the Commission Tripartite de la Direction des Eaux et Forêts (Permit No 47 and 215 18/MEF/SG/DGF/DSAP/SCB.Re).

**Table 4:** Description of the individuals included into the dataset.

Abbreviation	Group	Sex	Age	CSI
Le	A	F	Adult	0.53
Is	A	F	Adult	0.92
Lu	A	M	Adult	1.05
Pa	A	M	Adult	1.49
Th	A	M	Sub-adult	83.84
Bo	B	F	Adult	0.27
Al	B	F	Adult	11.32
Ri	B	F	Adult	26.53
Ba	B	M	Adult	0.70
Om	B	M	Adult	0.71
Ti	B	M	Adult	0.79
Ja	B	M	Sub-adult	51.52
To	F	F	Adult	0.94
Lu	F	F	Adult	283.79
Ma	F	F	Adult	3.27
Ca	F	M	Adult	0.35
Ju	F	M	Adult	1.54
Co	J	F	Adult	19.00
Ca	J	F	Adult	192.70
Sy	J	F	Adult	393.59
Pa	J	M	Adult	0.57
Ta	J	M	Adult	0.78
Mo	J	M	Adult	126.97
Ku	J	M	Adult	69.09
Ka	J	M	Sub-adult	61.67

### Data collection

Between May to July (later referred to as mating season) and September to November (later referred to as birth season), data were collected by focal scent mark observations^102^. Scent-marking behaviour were observed *ad libitum*^102^ during 27 to 34 half-days in each group. During these sessions, a total of 120 scent-marking behaviour (26 to 34 per group) served as foci for 15 min observations that were video recorded. During these 15 min observation periods, we annotated each individual passing the focal scent-marking spot, its identity, whether it performed scent marking or not, the date, the time, the context and the identity of all the other individuals present in the radius of 3, 5 and 10 m. If an individual scent marked not directly on the original scent-marking spot but on one in close proximity, we also considered it in our analysis and took previous pass-by events on this spot into account. The context was classified using four categories: resting, feeding, travelling and disturbance defining the group activity. The context 'disturbance' referred to situations in which individuals of the group are vigilant, and none of the other three context categories could be attributed to the situation. Cases when individuals of another group were visible were excluded.

Additionally, from May to November 2018, we also carried out 30 min individual focal observations in the morning between ca. 07:00–10:00 h and afternoon between14:00–17:00 h. A given individual was never observed for more than one 30 min session per day, and observations were balanced among each observation hour for each individual. The final dataset included 367 h of focal observations, with an average of 14.7 h per individual and was used to calculate the social values of the individuals and dyads.

### Data analyses

All analyses were carried out using R (version 3.6.0)^103^ and RStudio (version 1.2–1335)^104^.

#### Social values of individuals

We calculated the CSI (Composite Sociality Index^70^; Eq. (1)) for each individual based on three mutually exclusive affiliative behaviours: body contact, grooming and huddling. For each individual, we first calculated the proportion of time spent in body contact, huddling and grooming with an individual of its group (except juveniles). The resulting hourly rates for each of the three behaviours (${{{r}}.{{b}}{{c}}}_{i},{{{r}}.{{h}}{{u}}}_{{{i}}},{{{r}}.{{g}}{{r}}}_{{{i}}}$) were next divided by the respective mean rate for the group of the given individual before being summed up. To obtain the CSI, the summed value was divided by three, corresponding to the number of behaviours considered. We further attributed to each individual a CSI rank within each group and age-sex category, with individuals of rank 1 being the ones interacting the most often. To obtain the difference in CSI between two individuals we subtracted the CSI value of the individual in the audience to the CSI value of the focal. These CSI difference values were scaled using the R function 'scale' within each group.

1 $${{{C}}{{S}}{{I}}}_{{{i}}}=\frac{\frac{{{{r}}.{{b}}{{c}}}_{{{i}}}}{{{{m}}{{e}}{{a}}{{n}}({{r}}.{{b}}{{c}})}_{{{g}}{{r}}{{o}}{{u}}{{p}}}}+\frac{{{{r}}.{{h}}{{u}}}_{{{i}}}}{{{{m}}{{e}}{{a}}{{n}}({{r}}.{{h}}{{u}})}_{{{g}}{{r}}{{o}}{{u}}{{p}}}}+\frac{{{{r}}.{{g}}{{r}}}_{{{i}}}}{{{{m}}{{e}}{{a}}{{n}}({{r}}.{{g}}{{r}})}_{{{g}}{{r}}{{o}}{{u}}{{p}}}}}{3}$$

#### Social values of dyads

We calculated the DSI (Dyadic Composite Sociality Index^71^; Eq. (2)) of each dyad of individuals in a given group (excluding juveniles) following the same principle as for the CSI. Because two individuals were never observed simultaneously in a given group, interaction rates for a given dyad A-B could be calculated by summing up the rates associated with A being focal and interacting with B and B being focal and interacting with A. For each individual, we first calculated the time spent in body contact, huddling and grooming with each of its adult group members and divided it by the total observation duration of this individual while its partner was present in the group. We further attributed to each dyad a DSI rank within each group and age-sex category, with dyads of rank 1 being the most social dyads of their group. We used rank instead of raw DSI as we were not interested in group differences. In this way, the most social dyad of each group is attributed with the same social value.

2 $${{{D}}{{S}}{{I}}}_{{{d}}}=\frac{\frac{{{{r}}.{{b}}{{c}}}_{{{d}}}}{{{{m}}{{e}}{{a}}{{n}}({{r}}.{{b}}{{c}})}_{{{g}}{{r}}{{o}}{{u}}{{p}}}}+\frac{{{{r}}.{{h}}{{u}}}_{{{d}}}}{{{{m}}{{e}}{{a}}{{n}}({{r}}.{{h}}{{u}})}_{{{g}}{{r}}{{o}}{{u}}{{p}}}}+\frac{{{{r}}.{{g}}{{r}}}_{{{d}}}}{{{{m}}{{e}}{{a}}{{n}}({{r}}.{{g}}{{r}})}_{{{g}}{{r}}{{o}}{{u}}{{p}}}}}{3}$$

#### Estimation of the audience effect on anogenital scent marking

For a given individual, we only considered anogenital marking events that occurred with a time-lapse of at least 5 min between each other. We selected passing events (without scent marking) on the same criteria. We included only individuals for whom we had at least two observations of each passing and marking. Three males that emigrated during the period had to be excluded because we had only one observation of either passing or marking. The final male dataset included 14 individuals (3 sub-adults and 11 adults) observed for 60 pass events and 105 anogenital marking events. The female dataset included 11 adult females observed for 44 pass-by and 118 scent-marking events.

We first fitted two independent Generalized Linear Mixed Models (GLMM) for both sexes, estimating the influence of the audience composition on the probability of anogenital-marking behaviour to occur at a given time. These models had a binomial error structure and logit link function^105^ and were run for each audience radius. These models were fitted using the function glmer of the R package lme4 (version 1.1–21)^106^ with the optimiser' 'bobyqa'. As fixed effect, we included the proportions of males and adult females present in the given distance radius. To control for age (for males only as we only had one age class for females), context and season we also included these terms in the model as control predictors. Individual identity and date were included as random factors to account for individual variations and the possible effect of particular events.

To reduce the risk of type I errors^107^, we included all possible random slopes components (the proportion of males, the proportion of adult females, context and season within individual identity). We manually dummy-coded and centred context, season and age, and z-transformed the proportion of males and the proportion of females before including them as random slopes. Initially, we also included all correlations among random intercepts and slopes for all models. However, for females, these were all estimated to have absolute values being essentially one indicating that they were not identifiable^108^. Hence, we removed these correlations from the female model.

As an overall test of the effect of audience composition on the probability to anogenital scent mark, we compared the full model with the null model lacking the fixed effects characterising the audience (proportion of males and proportion of females) but comprising the control fixed effects and the same random effect structure as the full model^107^. This comparison was performed using a likelihood ratio test^109^.

Model stability was assessed by comparing the estimates of the model run on the full dataset with the ones run on datasets, excluding each level of the random effects one after the other^110^. The models were relatively stable (for males: Supplementary File 1.A; for females: Supplementary File 1.C). To control for potential collinearity problems, we calculated the Variance Inflation Factors^111^ for the model, excluding the random effects. VIF values ranged from 1.03 to 1.76 for the males (Supplementary File 1.B) and from 1.07 to 2.20 for females (Supplementary File 1.D). To control for multiple testing, we corrected the p-values using the p.adjust function with a Bonferroni method.

Confidence intervals were derived using the function bootMer of the package lme4, using 1,000 parametric bootstraps and bootstrapping over the random effects, too (argument' use.u' set to TRUE). Tests of the individual fixed effects were derived using likelihood ratio tests^112^ (R function drop1 with argument' test' set to" Chisq"). We determined the proportion of the total variance explained by the fixed effects (R^2^_m_; marginal coefficient of determination), and the proportion of the variance explained by both fixed and random effects (R^2^_c_; conditional coefficient of determination) following the method recommended by Nakagawa et al.^113^ and using the function r.squaredGLMM of the package MuMIn (version 1.43.6)^114^. Because our models seem to suffer singularity issues, we further applied a Bayesian method as recommended by the authors of the "lme4" package^106^. This approach should allow both regularising the model via informative priors and giving estimates and credible intervals for all parameters that average over the uncertainty in the random-effects parameters. Details on the methods and outputs of these models are provided in supplementary material (Supplementary File 2).

To account for the nonindependence of individuals within a group and the network structure of their interactions, we first used valued exponential random graph models (ERGM)^115^ to understand how the nature of the audience may influence the probability of anogenital marking. We implemented an ERGM based on a directional weighted matrix corresponding to the number of observed anogenital marking events of a focal individual (tail) when a given individual of its group was in the audience (head).

Models were implemented with a Poisson reference distribution, and the term "sum" corresponding to the sum of the edge weights (equivalent to an intercept in a linear modelling scenario) was added to the model. In addition, a "nonzero" term was added to control for zero inflation in the distribution of edge weights. Moreover, because structural terms are essential for correct model specification^116,117^ we included a mutuality term (sum of the minimum edge weights for each potential edge), and a cyclical weights term allowing for exploring hierarchical structure^118^. Two terms were included as control predictors: an edge covariate term to account for the amount of time an individual was observed in the presence of a given individual in the audience^119^ and a node-level covariate term to control for the effect of the group. Moreover, an offset term was added to acknowledge the fact that we only consider intra-group interactions. The terms described so far were the terms remaining in the null-model.

As node level predictors, we included the interaction between sex (only for adults) and the CSI rank of the individual in the audience (in-edges). As edge covariates, we included the interaction between the sexes and the difference between the CSI of the focal individual and the individual in the audience and the interaction between the sexes and the DSI rank corresponding to the dyad in question. All the terms corresponding to the main effects and the dummy variables (with the exception of the reference male-male) were also included in the model.

ERGMs were implemented in R using the statnet suite of packages^115,120–123^. The code to implement this model is provided in the ESM, Supplementary File 3. We manually dummy coded and centred the sex interacting and z-transformed all the explanatory variables before including them into the model. The goodness of fit was assessed for each model by simulating 1000 networks and comparing the distribution of their coefficients to the observed coefficients^124,125^ (Supplementary Figs. 1, 2 and 3). MCMC diagnostics were used to assess ERGM convergence (“mcmc.diagnostics” function in the ergm package) (Supplementary Figs. 4, 5 and 6). To assess the overall test of the significance of the interaction between sex and sociality we compared the deviance of the full model to the deviance of the null model described above. This comparison was based on a likelihood ratio-test^107,109^, R function anova with the argument test set to "chisq". To test the significance of the individual interactions between sex and the three social variables, we compared the full model's deviance with that of a corresponding reduced model not comprising this interaction. To control for multiple testing, we corrected the p-values using the p.adjust function with a Bonferroni method. Confidence intervals for the interaction effects were obtained by bootstrapping the response matrix (adding or subtracting 1 to an intra-group edge weight).

### Ethics declarations

All applicable international, national, and/or institutional guidelines for the care and use of animals were followed. The authors complied with the ARRIVE guidelines^100^. This study adhered to the Guidelines for the Treatment of Animals in Behavioral Research and Teaching^101^ and the legal requirements of the country (Madagascar) in which the work was carried out. The protocol for this research was approved by the Commission Tripartite de la Direction des Eaux et Forêts (Permit No 47 and 215 18/MEF/SG/DGF/DSAP/SCB.Re).

## Supplementary Information

Supplementary Figure S1.

Supplementary Figure S2.

Supplementary Figure S3.

Supplementary Figure S4.

Supplementary Figure S5.

Supplementary Figure S6.

Supplementary Information 1.

Supplementary Information 2.

Supplementary Information 3.

## Data Availability

The datasets generated and analysed during the current study are available from the corresponding author on reasonable request.
