# Supplementary material for: Sex differences in audience effects on anogenital scent marking in the red-fronted lemur
Source: Sci Rep. 2022 Mar 28;12:5266. doi: 10.1038/s41598-022-08861-2 (PMC8960772; doi:10.1038/s41598-022-08861-2)

**Supplementary Figure S2:** goodness of fit for the exponential random graph model on the audience effect on scent-marking in redfronted lemurs when considering a 5m radius. For each model term, the estimate of the model (blue lines) is compared to the distribution of the estimates of 1000 simulated networks. This figure was visualized and edited using R (https://www.r-project.org/).


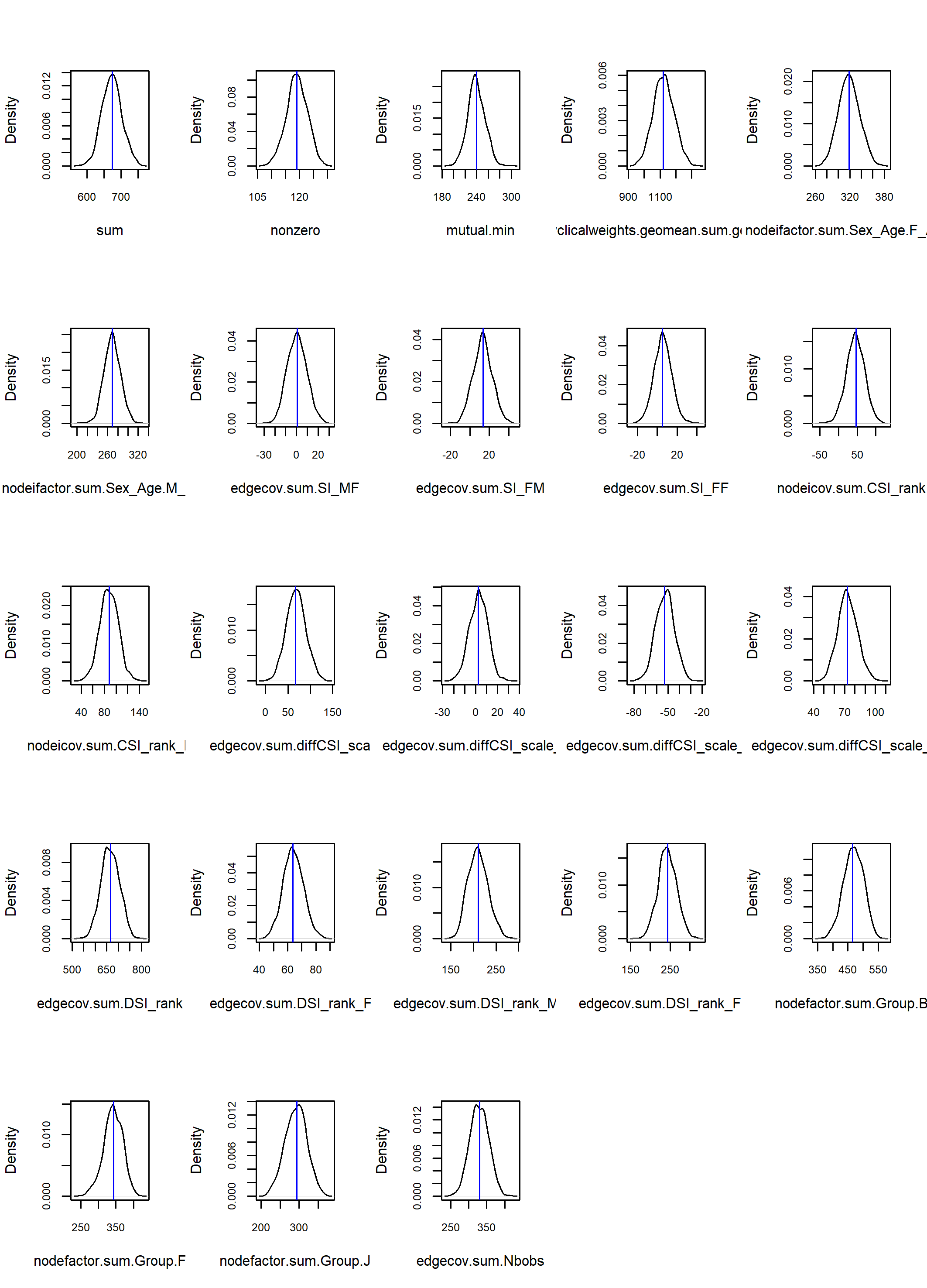

Supplement: Supplementary file 2 — Supplementary Figure S2. [file 41598_2022_8861_MOESM2_ESM.docx]
