# Supplementary material for: Sex differences in audience effects on anogenital scent marking in the red-fronted lemur
Source: Sci Rep. 2022 Mar 28;12:5266. doi: 10.1038/s41598-022-08861-2 (PMC8960772; doi:10.1038/s41598-022-08861-2)

**Supplementary Figure S3:** goodness of fit for the exponential random graph model on the audience effect on scent-marking in redfronted lemurs when considering a 10m radius. For each model term, the estimate of the model (blue lines) is compared to the distribution of the estimates of 1000 simulated networks. This figure was visualized and edited using R (https://www.r-project.org/).


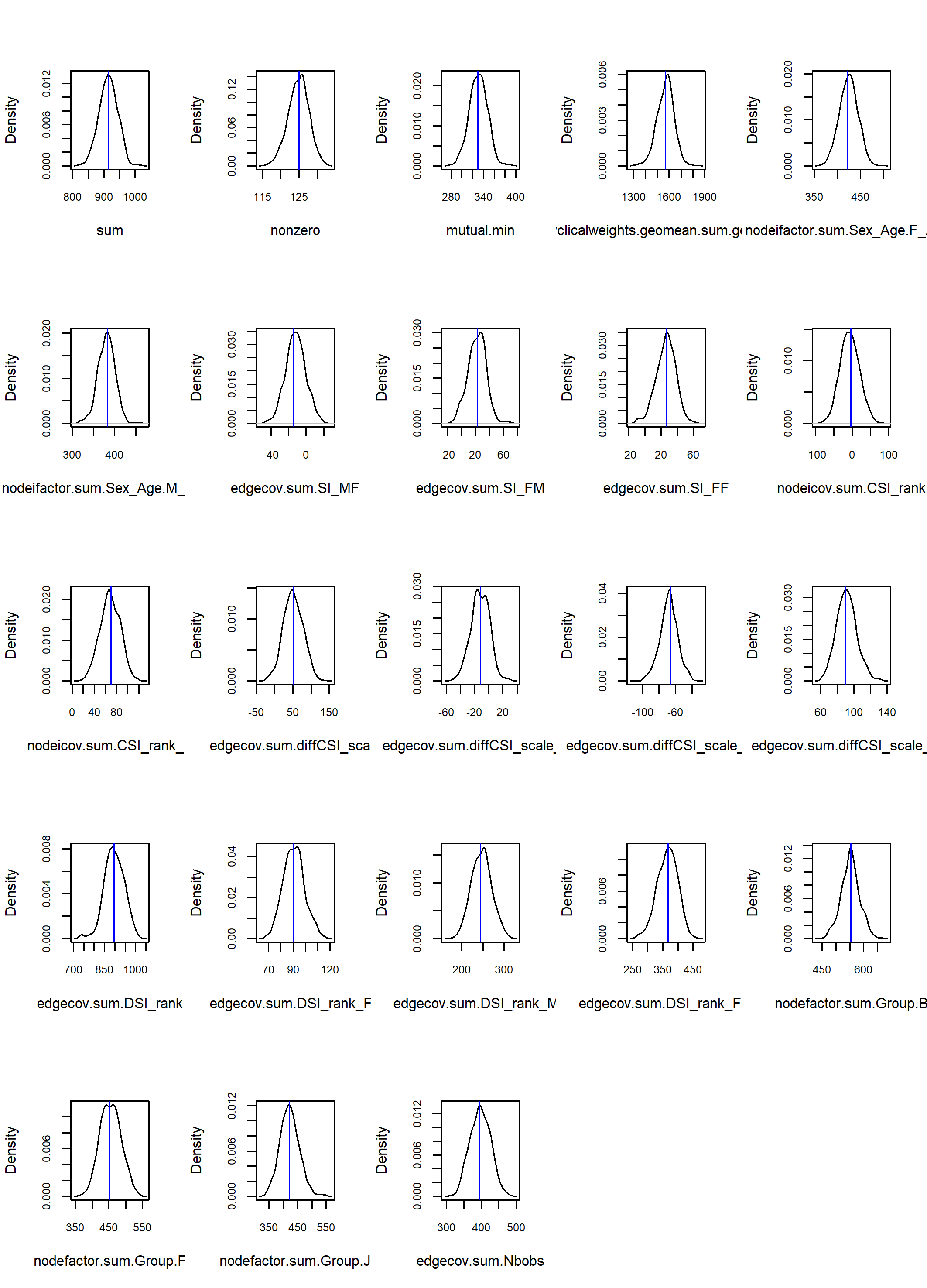

Supplement: Supplementary file 3 — Supplementary Figure S3. [file 41598_2022_8861_MOESM3_ESM.docx]
