# Supplementary material for: Sex differences in audience effects on anogenital scent marking in the red-fronted lemur
Source: Sci Rep. 2022 Mar 28;12:5266. doi: 10.1038/s41598-022-08861-2 (PMC8960772; doi:10.1038/s41598-022-08861-2)

**Supplementary Figure S5:** MCMC diagnostics for the exponential random graph model on the audience effect on scent-marking in redfronted lemurs when considering a 5m radius. For each model term, in the first column figures illustrate Markov chains for the parameter, α; in the second column are given posterior distributions for α. This figure was visualized and edited using R (https://www.r-project.org/).


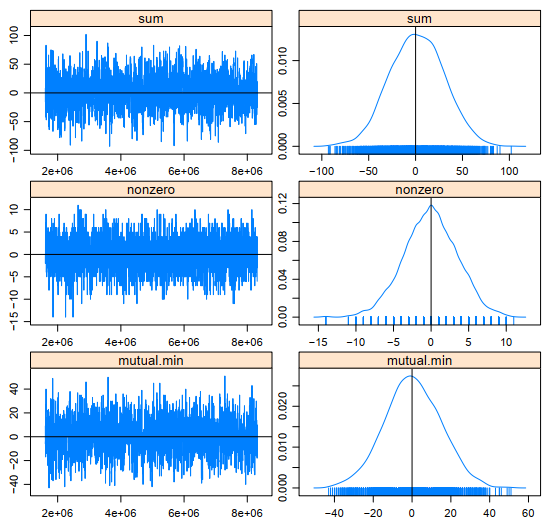


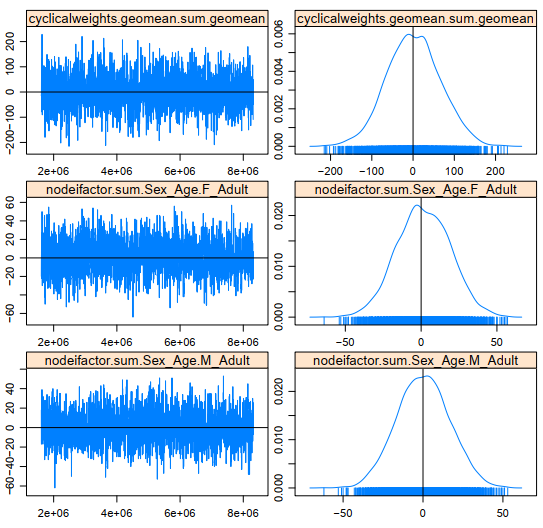


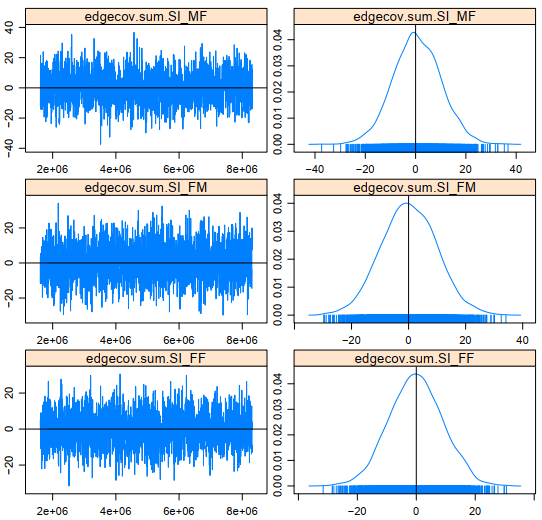


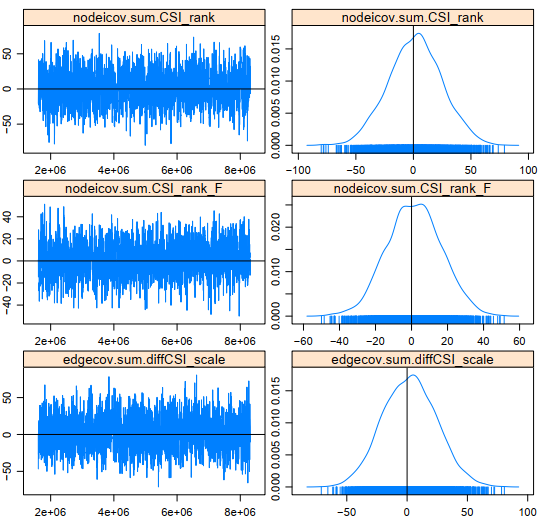


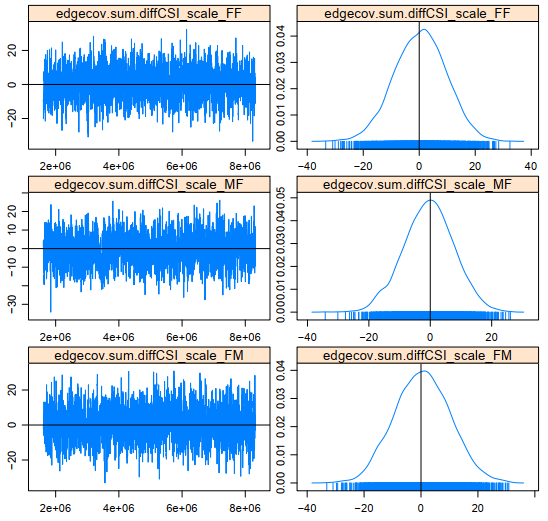


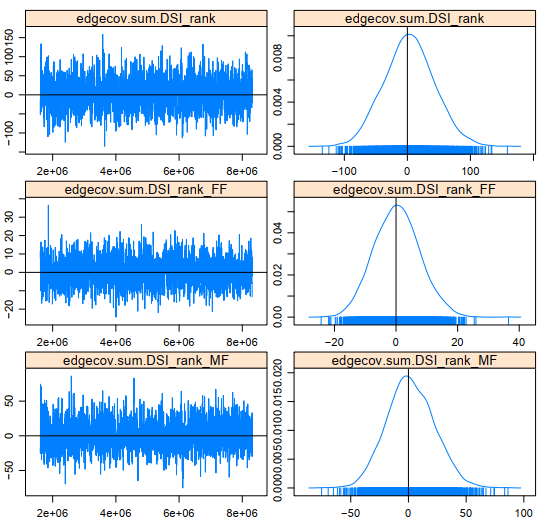


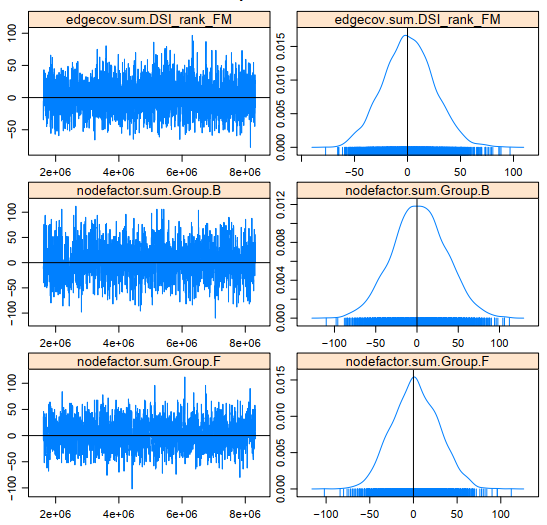


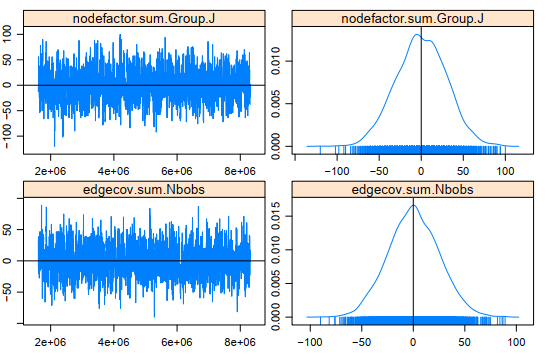

Supplement: Supplementary file 5 — Supplementary Figure S5. [file 41598_2022_8861_MOESM5_ESM.docx]
