# Supplementary material for: Sex differences in audience effects on anogenital scent marking in the red-fronted lemur
Source: Sci Rep. 2022 Mar 28;12:5266. doi: 10.1038/s41598-022-08861-2 (PMC8960772; doi:10.1038/s41598-022-08861-2)
Supplement: Supplementary file 7 — Supplementary Information 1. [file 41598_2022_8861_MOESM7_ESM.docx]

**Supplementary File S1:** Diagnostics for the 6 generalised linear mixed models

Supplementary File S1.A. Stability of the estimates for the male models when considering a) the 3m radius, b) the 5m radius, and c) the 10m radius. This figure was visualized and edited using R (https://www.r-project.org/).

a.


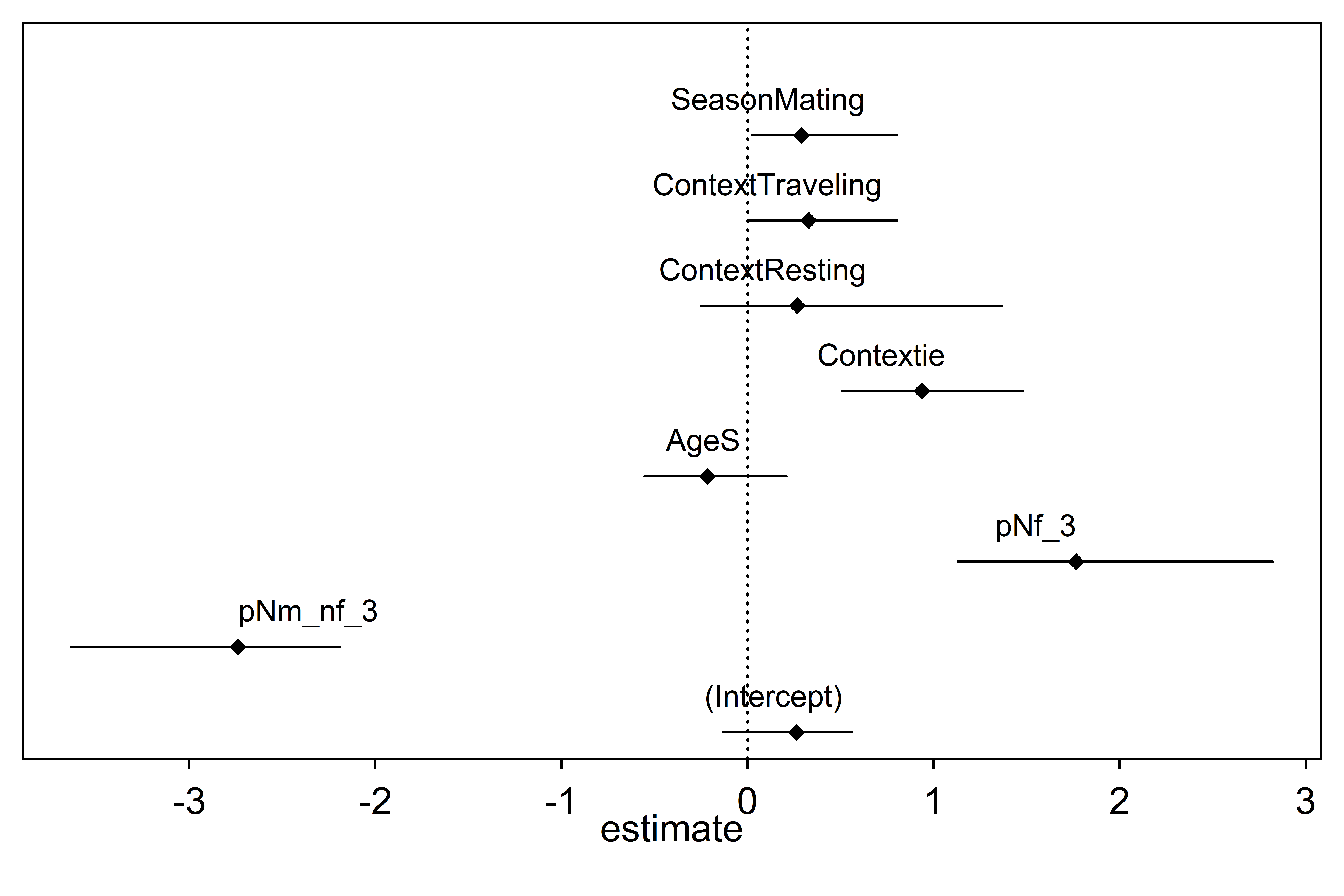


b.


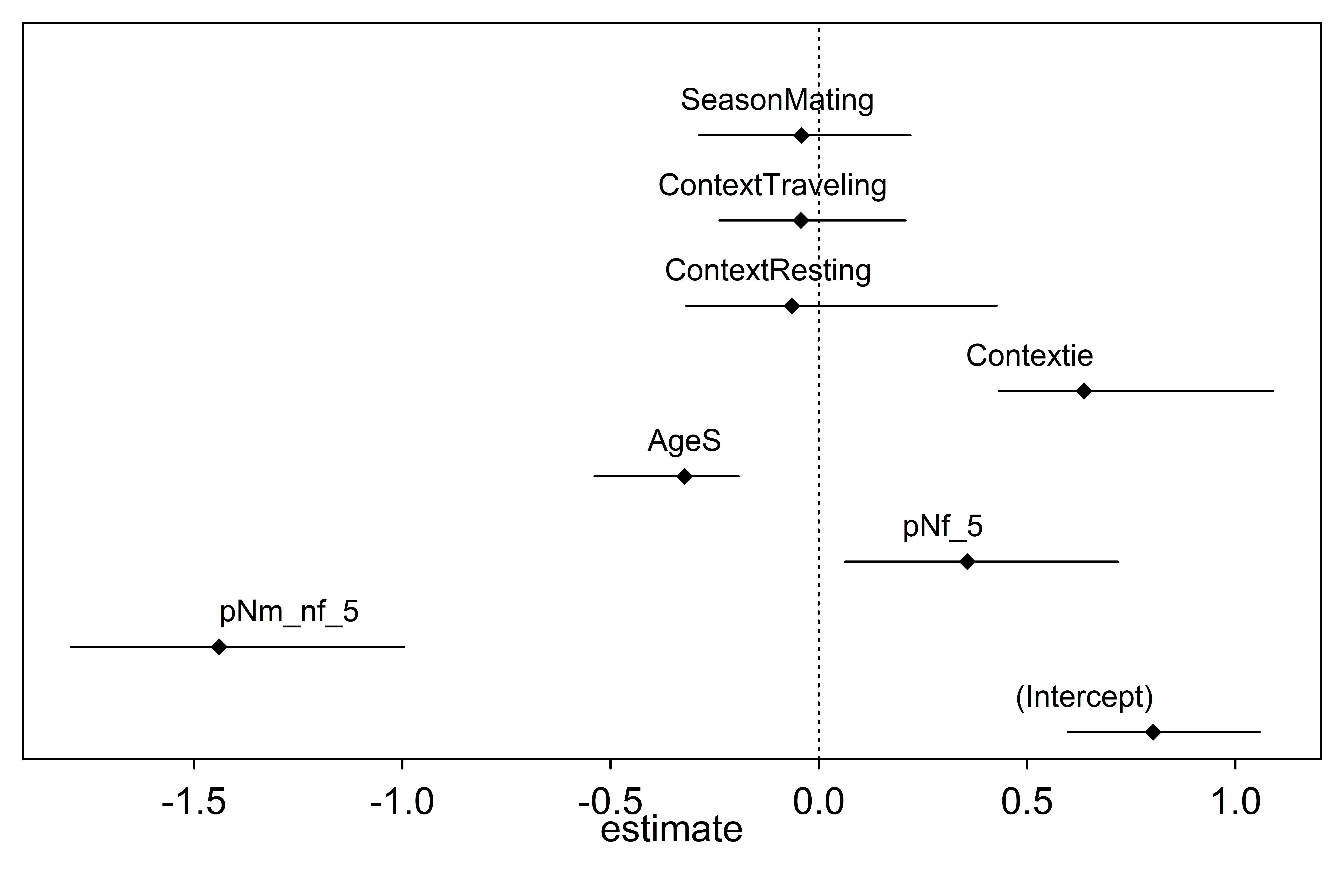


c.


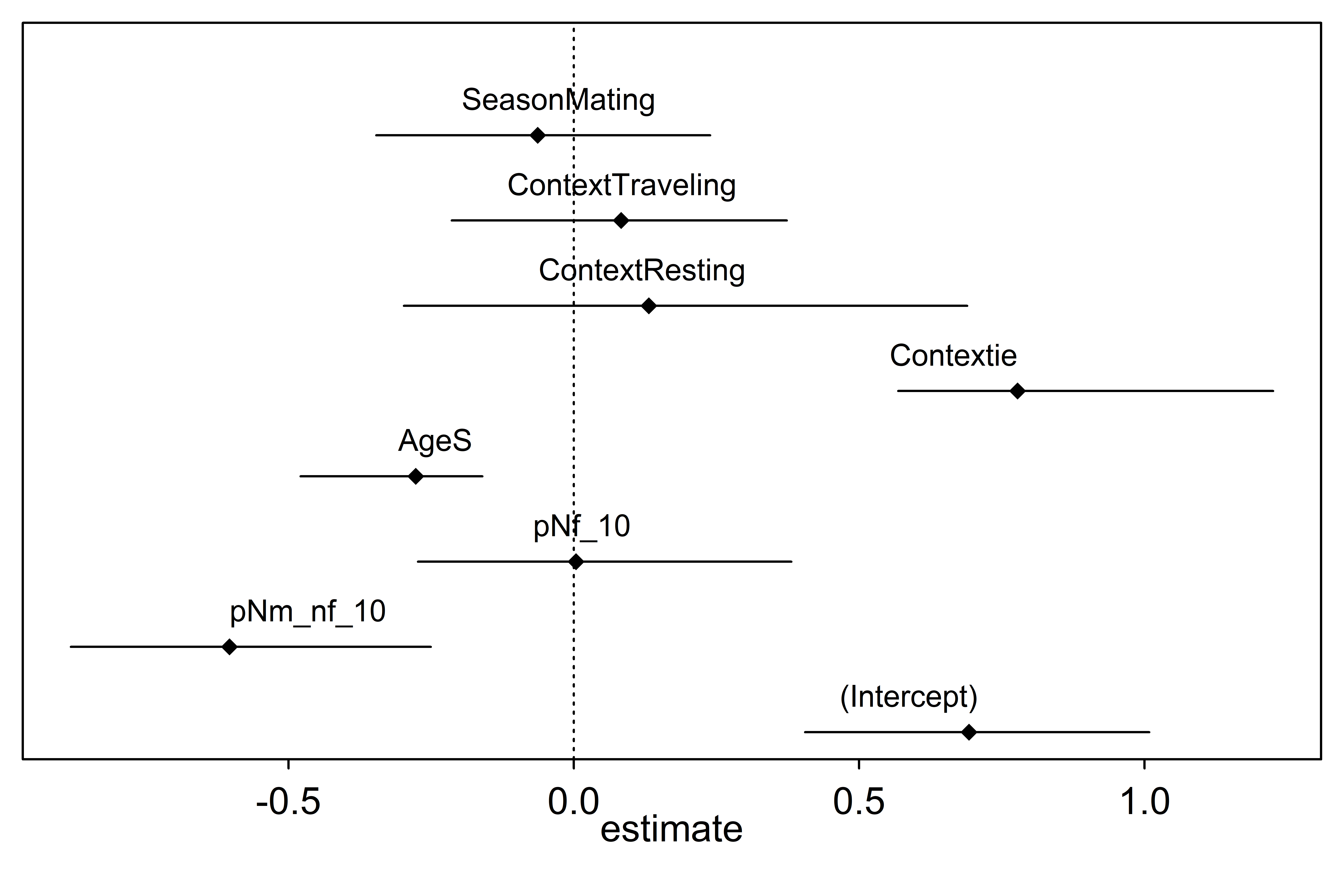


Supplementary File S1.B. Variation Inflation Factors for the male models.

|  | 3m | 5m | 10m |
| --- | --- | --- | --- |
| Proportion of females | 1.35 | 1.54 | 1.76 |
| Proportion of males | 1.15 | 1.27 | 1.40 |
| Age | 1.04 | 1.03 | 1.04 |
| Context | 1.07 | 1.13 | 1.11 |
| Season | 1.13 | 1.09 | 1.04 |

Supplementary File S1.C. Stability of the estimates for the female models when considering a) the 3m radius, b) the 5m radius, and c) the 10m radius. This figure was visualized and edited using R (https://www.r-project.org/).

a.


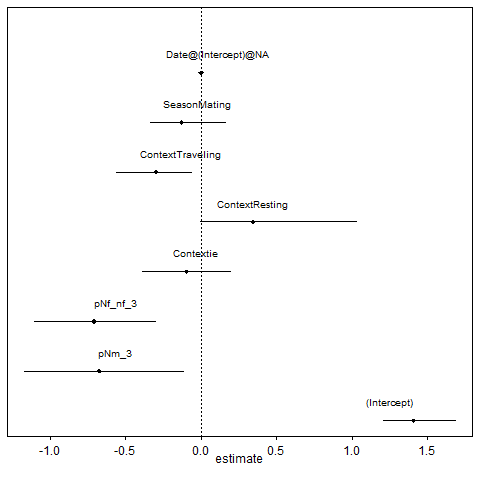


b.


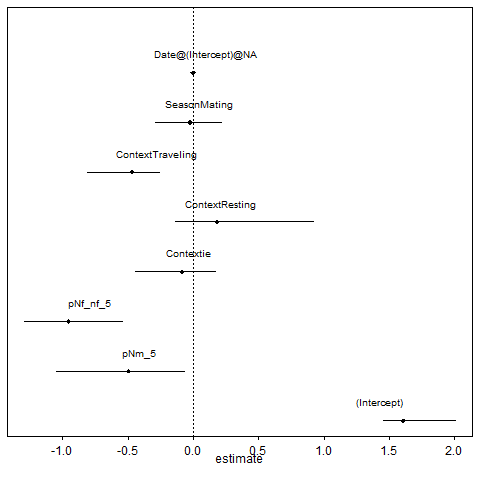


c.


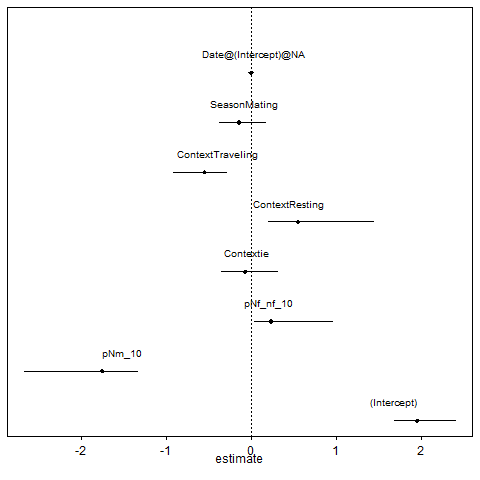


Supplementary File S1.D. Variation Inflation Factors for the female models.

|  | 3m | 5m | 10m |
| --- | --- | --- | --- |
| Proportion of females | 2.12 | 2.20 | 1.80 |
| Proportion of males | 2.03 | 2.25 | 1.83 |
| Context | 1.07 | 1.09 | 1.11 |
| Season | 1.08 | 1.09 | 1.07 |
